# On the Role of Microstructure and Defects in the Room and High-Temperature Tensile Behavior of the PBF-LB A357 (AlSi7Mg) Alloy in As-Built and Peak-Aged Conditions

**DOI:** 10.3390/ma16072721

**Published:** 2023-03-29

**Authors:** Lavinia Tonelli, Erica Liverani, Gianluca Di Egidio, Alessandro Fortunato, Alessandro Morri, Lorella Ceschini

**Affiliations:** Department of Industrial Engineering (DIN), Alma Mater Studiorum—University of Bologna, Viale del Risorgimento 2, 40136 Bologna, Italy

**Keywords:** additive manufacturing, aluminum alloys, structural integrity, microstructure, mechanical properties, heat treatment

## Abstract

Additive processes like Laser Beam Powder Bed Fusion (PBF-LB) result in a distinctive microstructure characterized by metastability, supersaturation, and finesse. Post-process heat treatments modify microstructural features and tune mechanical behavior. However, the exposition at high temperatures can induce changes in the microstructure. Therefore, the present work focuses on the analyses of the tensile response at room and high (200 °C) temperature of the A357 (AlSi7Mg0.6) alloy processed by PBF-LB and subjected to tailored T5 (direct aging) and T6R (rapid solution treatment, quenching, and aging) treatments. Along with the effect of microstructural features in the as-built T5 and T6R alloy, the role of typical process-related defects is also considered. In this view, the structural integrity of the alloy is evaluated by a deep analysis of the work-hardening behavior, and quality indexes have been compared. Results show that T5 increases tensile strength at room temperature without compromising ductility. T6R homogenizes the microstructure and enhances the structural integrity by reducing the detrimental effect of defects, resulting in the best trade-off between strength and ductility. At 200 °C, tensile properties are comparable, but if resilience and toughness moduli are considered, as-built and T5 alloys show the best overall mechanical performance.

## 1. Introduction

Hypoeutectic Al-Si-Mg conventional cast alloys are widely used in the automotive industry, especially for manufacturing lightweight structural castings that often require complex designs. AlSi7Mg alloys (i.e., A356, A357, and their variations), in particular, are recommended for applications in the power train system (e.g., engine blocks, cylinder heads) due to the high strength and elongation at room temperature achievable after dedicated heat treatment (T6, consisting of solution treatment, quenching, and aging) that promote strengthening due to the precipitation of Mg-Si phases during aging [1,2]. However, for these specific applications, it is crucial to guarantee a high strength at elevated temperatures (up to 200–250 °C) that engine components can commonly experience during their service life [3]. Conventional cast A356 or A357 T6 alloys suffer a non-negligible decrease in mechanical strength if exposed to temperatures close to 200 °C due to coarsening of reinforcing phases related to over-aging [4]. For high-temperature applications, alloys containing a certain amount of Cu assure better thermal stability [4,5]. However, the effect of thermal exposure on the microstructure and mechanical properties of AlSi7Mg alloys processed by innovative additive manufacturing technologies, like laser-based powder bed fusion (PBF-LB), is currently lacking. In recent years, literature has proved that Al-Si-Mg alloys, mostly AlSi10Mg and AlSi7Mg, can be successfully processed with PBF-LB [6,7,8]. PBF-LB is based on selective melting, layer after layer, of a bed of fine metallic powder and enables the manufacturing of extremely complex designs with high accuracy. For these reasons, in the automotive field and in the context of promoting more sustainable mobility, PBF-LB can effectively enhance the mass reduction of vehicles by lightweight structural components. In this view, mechanical characterization, even at a temperature consistent with the service life, has to be performed.

From the material point of view, it is worth mentioning that the microstructure resulting from the PBF-LB process is in the unique condition of being supersaturated, metastable, and extremely fine [8]. Consequently, strengthening mechanisms differ substantially from conventional cast alloys and are mostly related to the solid solution, microstructure refinement, and, to a lesser extent, precipitation hardening [9]. Therefore, it is crucial to assess mechanical behavior and its correlation to microstructural features and heat treatment conditions, even when exposed to high temperatures. Furthermore, innovative heat treatments can be optimized on this peculiar microstructure. For example, artificial aging performed directly from the as-built condition promotes the precipitation from the supersaturated solid solution (T5 treatment) [10,11,12,13,14,15].

Moreover, conventional T6 heat treatment variations can be optimized to induce microstructure homogenization [10,11,12,13,14,16,17]. On the other hand, PBF-LB suffers from typical microstructural defects, such as lack of fusion region, gas, or keyhole porosity, that can also affect the mechanical behavior of the alloy [18]. The role of heat treatment in defining the mechanical response of the A357 PBF-LB alloy has been widely investigated in the literature [10,11,12,13,14,15,16]. Some works focused on the correlation between mechanical properties and defects content for the Al-Si PBF-LB alloys can also be found [19,20]. However, the concurrent effect of defects content and heat treatment condition must be addressed. Furthermore, even though a few attempts to assess the high-temperature behavior of AlSi10Mg PBF-LB alloy have been carried out [21,22,23,24] to the best of the authors’ knowledge, mechanical properties at high temperatures of the A357 PBF-LB alloy have yet to be investigated.

Based on the above, this study aims at (i) investigating tensile behavior at room and high (200 °C) temperature of the A357 PBF-LB in the as-built state and after tailored heat treatments (T5 and T6, optimized in previous work [25]) at peak-aging condition; (ii) correlating tensile behavior to both microstructural features, induced by heat treatments, and defects, induced by the process, by performing the analysis of structural integrity of the alloy with the support of microstructural and fractographic observations.

## 2. Materials and Methods

### 2.1. Samples Production and Heat Treatments

Tensile AlSi7Mg0.6 (designated as A357 according to EN1780 standard [26]) samples were produced by PBF-LB, starting from atomized powder supplied by Carpenter Additive (Carpenter Additive, Carpenter Technology Corporation, Philadelphia, PA, USA). Powder chemical composition is reported in Table 1, and its complete characterization can be found in a previous work [27]. In Table 1, the nominal chemical composition of powder was compared to one of the printed samples and checked with a Glow Discharge Optical Emission Spectroscopy (GDOES, GDA-650 Spectrum Analytik GmbH, Hof, Germany). The chemical composition of powder and printed samples satisfied the requirements of the EN 1706 standard for the AlSi7Mg0.6 alloy [28]. More importantly, no deviation in the Mg content was verified in the PBF-LB samples, suggesting that no vaporization of this low melting element occurred during the process.

**Figure 1:**
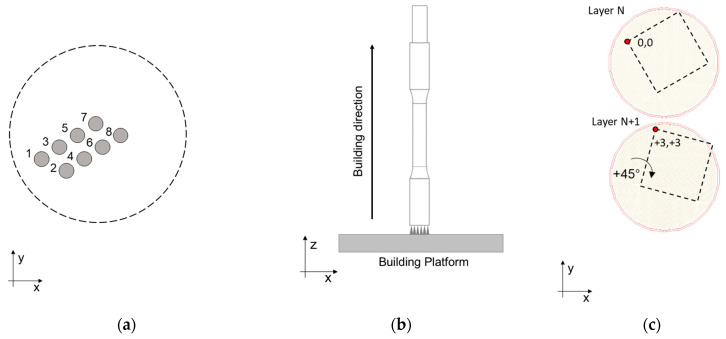
Tensile samples manufacturing: (**a**) printing position and scanning order; (**b**) building direction, (**c**) chessboard scan strategy.

A total of 24 samples were arranged in four identical building platforms of 8 specimens each to ensure an equivalent in-process thermal cycle. Platforms were designed to enhance the quality of printed parts that, in the absence of pre-heating as in this case, requires limiting the cooling of samples between two subsequent layers (Figure 1a).

Before each job, A357 powder was subjected to a drying pre-treatment, optimized in a previous work [27], at 60 °C for 3 h. A SISMA MySint 100 RM system (SISMA S.p.a, Piovene Rocchette, Italy), equipped with a 200 W fiber laser with a spot diameter of 55 μm, was used for sample fabrication. The process occurred in a nitrogen environment with residual oxygen content less than 0.1 vol.% and, as previously mentioned, without using a pre-heated platform. Supports, process parameters, and scanning strategy were designed by dedicated software (AutoFab RnD 2.0, Marcam Engineering GmbH, Bremen, Germany) and summarized in Table 2. In addition, process parameters in Table 2 were elaborated based on a previous study [27].

**Figure 2:**
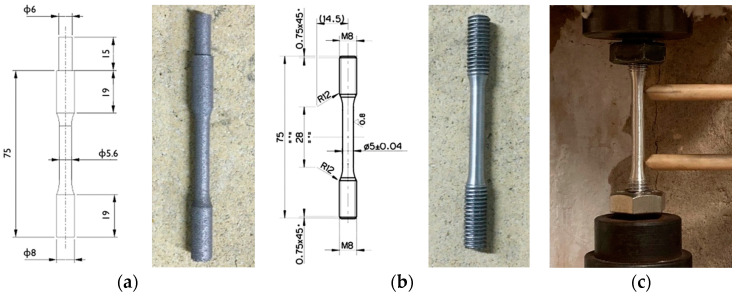
Tensile samples geometry and dimensions: (**a**) near-net-shape sample obtained by PBF-LB process, (**b**) final sample after machining. In (**c**): equipment for tensile tests.

Samples were printed along the direction perpendicular to the platform (along the *z*-axis, Figure 1b), and a roto translating 3 × 3 mm^2^ chessboard scanning strategy (Figure 1c) with an enabled skywriting function was utilized. Round cross-section tensile samples were built with a near-net-shape geometry (Figure 2a) and then machined to final dimensions (Figure 2b); the gripped end geometry was designed to facilitate post-process machining. Tensile samples were proportional (as defined by EN ISO 6892-1 [30]) with a 5 mm final diameter, a 28 mm parallel length, and a 25 mm original gauge length.

Among the 24 produced samples, 8 were tested in the as-built condition, while 16 were subjected to a post-process heat treatment. Direct artificial aging from the as-built condition (T5 temper) and innovative treatment involving a rapid solution treatment, quenching, and artificial aging (T6R temper) was performed [31]. Heat treatment parameters at peak-aged conditions (Table 3) have been tailored to the characteristic microstructure resulting from the PBF-LB process, which diverges substantially from conventional cast alloys. Heat treatment conditions were optimized in previous work [25] and were performed before machining to the final geometry.

### 2.2. Tensile and Hardness Tests

Tensile tests were conducted at room and high (200 °C) temperature, following the EN ISO 6892-1 and EN ISO 6892-2 standards [30,32]. Tests were performed on a screw tensile testing machine with a resistance furnace, a 20 kN load cell, and a clip-on extensometer (Figure 1c). Tests were conducted in displacement control mode, with a crosshead speed of 0.007 mm/s. A data acquisition system continuously acquired force and displacement values to compute engineering curves during the tests. All heat treatment conditions were tested at both temperatures, as evidenced in Table 3, and four repetitions were performed for each condition. A soaking time of 30 min was adopted for high-temperature tests to homogenize the sample temperature before the test. The temperature of 200 °C was chosen based on the service life of power-train components and on the threshold temperature at which microstructural coarsening of conventional cast alloys occurs [3,4].

According to the aforementioned standards [30,32], Young modulus (E) is evaluated based on force-extension curves as the slope of the linear portion of the curve, while yield stress is determined as proof strength at 0.2% strain (Rp_0.2_). The tensile strength is defined as Rm, and the percentage elongation after fracture (A%) is determined by deducing the elastic extension. The modulus of resilience (U_R_), defined as the area under the engineering stress-strain curve in the elastic region [33], and the modulus of toughness (U_T_), defined as the area under the whole engineering stress-strain curve [33], were evaluated for each sample tested. Furthermore, the true stress-true strain curves were computed [33,34]. For tests carried out at room temperature, the structural integrity of samples as a function of the temper condition was evaluated by a detailed analysis of the plastic region of the curve. The Voce equation and Kocks-Mecking model were obtained to describe the work-hardening behavior in as-built, T5, and T6R states. Evaluation of the structural integrity of samples was performed by computing quality indexes [35,36,37] and by comparing the experimental tensile strength to the target one that can be reached if no major microstructural discontinuities are present [36,38,39]. Moreover, the strain hardening exponent (n) was evaluated according to the ISO 10275 standard [40].

Sample hardness was evaluated following the Brinell method (HB10, with 2.5 mm ball diameter, 62.5 kgf force, and a force-diameter ratio equal to 10 [41]) before tensile tests and after the tests performed at 200 °C; the latter is defined as residual hardness.

### 2.3. Microstructural and Fractographic Characterization

After tensile tests, the surface fracture was analyzed at high magnification by a Field Emission Gun Scanning Electron Microscope (FEG-SEM, Tescan Mira 3) to investigate fracture morphology and mechanism.

Microstructural analyses were then devoted to correlate the static mechanical behavior to the microstructural features. To this aim, microstructural characterization was performed on sections extracted from the grip region of samples along the longitudinal (x-z) plane, parallel to the building direction, and along the transversal (x-y) plane, parallel to the building platform and consistent with the cross-section area. Metallographic sections were embedded in a conductive resin and then ground and polished to a mirror finish following standard procedures [42]. Microstructural features were revealed with chemical etching with Keller’s reagent (2.5 mL HNO_3_, 1.5 mL HCl, 1.0 mL HF and 95 mL distilled water [43]), performed by a 20 s immersion at ambient temperature. Microstructural investigation of etched sections was carried out using optical (OM, Zeiss Axio Imager A1) and FEG-SEM microscopy. Quantitative evaluation of microstructural features (i.e., area% of Si-rich region, as will be later discussed) was conducted with the ImageJ software (National Institutes of Health, version 1.46r, Bethesda, Bethesda, MD, USA) [44].

## 3. Results and Discussion

### 3.1. Room Temperature Tensile Behavior: Effect of Temper Condition

**Figure 3:**
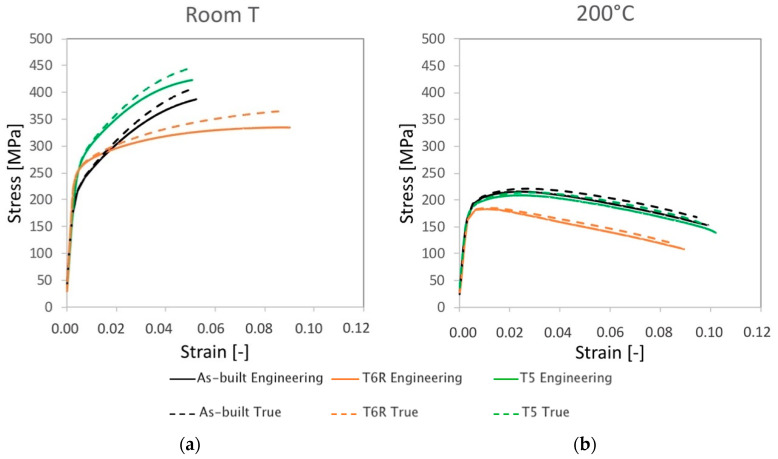
Representative engineering and true stress-strain curves for the as-built, T5, and T6R AlSi7Mg PBF-LB alloy: (**a**) room temperature and (**b**) high temperature (200 °C) tensile tests.

**Figure 4:**
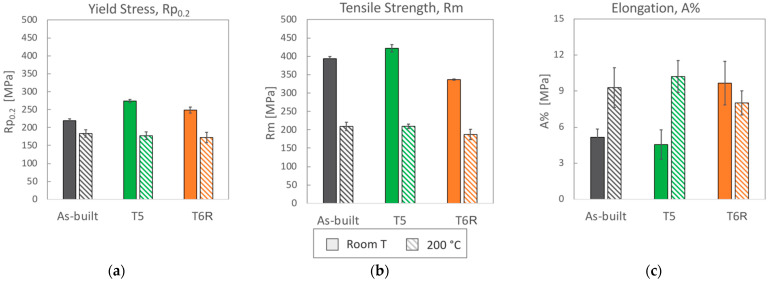
(**a**) Yield stress, (**b**) tensile strength, and (**c**) elongation to failure (mean value and standard deviation) obtained from tensile tests performed at room and high (200 °C) temperature for the as-built, T5, and T6R AlSi7Mg PBF-LB alloy.

**Figure 5:**
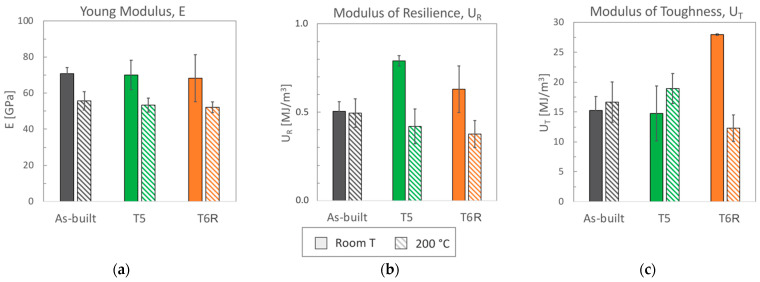
(**a**) Young, (**b**) resilience, and (**c**) toughness modulus (mean value and standard deviation) obtained from tensile tests performed at room and high (200 °C) temperature for the as-built, T5, and T6R AlSi7Mg PBF-LB alloy.

**Figure 6:**
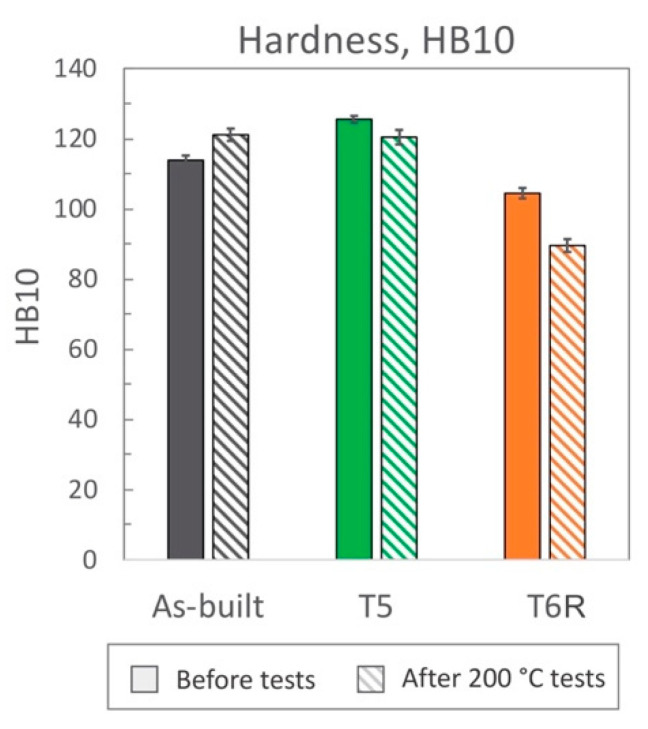
Hardness was evaluated on tensile samples after tests performed at room and high (200 °C) temperature for the as-built, T5, and T6R AlSi7Mg PBF-LB alloy (mean value and standard deviation).

Representative engineering and true stress-strain curves of the considered temper conditions (as-built, T5 and T6R) are compared in Figure 3a for room temperature tensile tests and Figure 3b for high temperature (200 °C) tests. Mechanical properties obtained from the analyses of the curves are reported in Figure 4 (yield stress, tensile strength, and elongation) and in Figure 5 (Young, resilience, and toughness modulus). The hardness of the alloy, measured after tensile tests, is reported in Figure 6.

By focusing on room temperature tests, a significant difference in tensile behavior can be noticed according to the temper condition of the alloy. Differences are related to elastic (yield stress, resilience modulus) and plastic (tensile strength, elongation, toughness modulus) properties, as confirmed by data in Figure 4 and Figure 5. Data are discussed mainly by focusing on mean values, as standard deviations for the given property were comparable among temper conditions. As regards yield stress, the as-built alloy was characterized by Rp_0.2_ = 216 ± 5 MPa and, regarding literature data [10,11,12,15,16,45], the value is comparable to samples fabricated in similar conditions (vertical building direction and no platform pre-heating), but it is about 30% lower than samples built with a pre-heated platform (temperatures range 100–150 °C). Presumably, samples built with platform pre-heating experienced artificial aging, thus justifying this difference [10,46]. Comparable considerations can also be drawn for ultimate tensile strength that, for the present work, was set at R_m_ = 394 ± 6 MPa. By also considering the good elongation to failure, equal to A% = 5.2 ± 0.7%, the alloy in the as-built state was characterized by an overall satisfying mechanical behavior.

Both T5 and T6R treatments increased the yield stress of the alloy that reached the highest value after the direct aging (T5) treatment. The yield stress increase compared to the as-built condition was +25% (274 ± 4 MPa) and +13% (249 ± 8 MPa) in the case of T5 and T6R, respectively. As discussed in the following, the increase in yield stress can be correlated to the precipitation of fine reinforcing particles, as evidenced in previous work [25]. An even higher increase was measured for the resilience modulus, which quantifies the material’s ability to adsorb energy when subjected to elastic deformation, equal to +56% for T5 (0.79 ± 0.03 MJ/m^3^) and +25% for T6R (0.63 ± 0.13 MJ/m^3^). By focusing on the plastic region of the stress-strain curves, the effect of the applied heat treatment diverged between T5 and T6R. T5 slightly increased (by 7%, reaching 422 ± 10 MPa) the tensile strength of the as-built alloy without dramatically affecting the elongation (equal to 4.6 ± 1.2%, decreased by 12%). As a result, the modulus of toughness, which quantifies the ability of the material to adsorb energy when subjected to plastic deformation without occurring into the fracture, was comparable for both conditions (15.3 ± 2.3 and 14.8 ± 4.6 MJ/m^3^ for as-built and T5, respectively). On the other hand, if compared to the as-built condition, the optimized T6R treatment induced a decrease in the tensile strength (equal to 337 ± 2 MPa, decreased by 14%) and a marked increase in the elongation (by 87%, reaching 9.7 ± 1.8%). Therefore, toughness was maximized by T6R treatment, which reached the value of 28 ± 0.13 MJ/m^3^ and increased by 83% compared to the as-built condition. It should be mentioned that toughness is an essential property for a mechanical structural component in all cases where, occasionally, during in-service conditions, the applied stress overcomes the yield. In line with the tensile results, when coming to hardness measurement (Figure 6), T5 treatment induced an increase (from 114 ± 1 of the as-built state to 126 ± 1 HB10). T6R treatment slightly decreased the alloy hardness, reaching 104 ± 1 HB10, even if it improved alloy ductility and the trade-off between strength and ductility.

**Figure 7:**
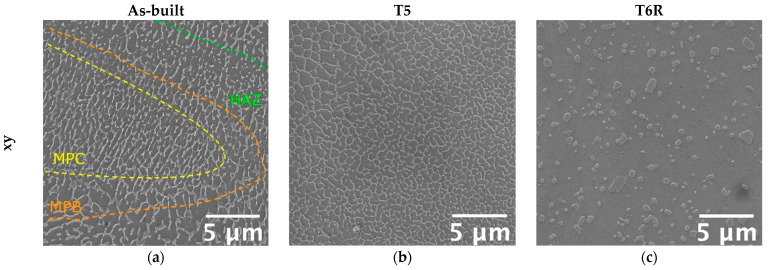

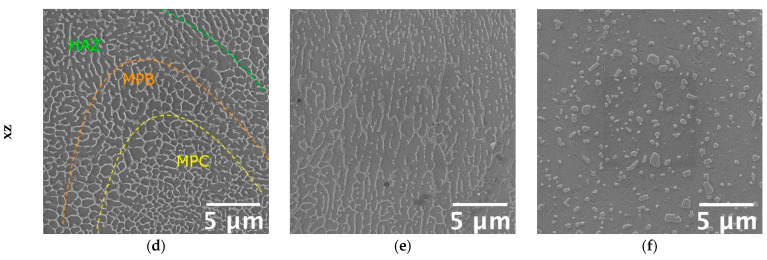
FEG-SEM high magnification micrographs showing the typical microstructure of the as-built, T5, and T6R AlSi7Mg PBF-LB alloy: (**a**–**c**) along the direction parallel to the building platform (xy plane); (**d**–**f**) along the direction parallel to the building one (xz plane).

Microstructural features determine mechanical properties, particularly the involved strengthening mechanisms. Therefore, a modification in the tensile behavior suggests a modification in the strengthening mechanisms, thus on microstructural features. It is well-known that a hierarchical arrangement characterizes the microstructure of PBF-LB Al-Si-Mg alloys, and several microstructural features, with a distinctive scale, can be resolved: (i) layer-by-layer structure formed by solidified melt pools with dimensions up to hundreds of micrometers, (ii) micrometric epitaxial grains passing over layers, (iii) sub-micrometric cellular substructure [8]. According to recent works [9,47,48,49,50,51,52], the cellular substructure has a crucial role in defining the mechanical properties of Al-Si-Mg alloys. Representative microstructure of as-built, T5, and T6R alloys, observed along the xy (parallel to the building platform) and xz (parallel to the building direction) planes, are reported in Figure 7. The figure shows the characteristic structure formed by sub-micrometric α-Al cells surrounded by a fine network of eutectic-Si. The as-built microstructure suffered from a certain degree of anisotropy. If analyzed in the xy plane, α-Al cells were almost equiaxed (Figure 7a); however, if observed in the xz plane, cells were elongated along the building direction (Figure 7d).

Furthermore, due to the repeated heat cycles experienced during the PBF-LB process, the morphology of the eutectic-Si network is not homogeneous. In particular, it changes according to the considered region of the melt pool formed due to the interaction between the laser beam and the powder bed [8,53]. If close to the core of the melt pool (MPC in the figure), the network is fine and interconnected; if close to the border (MPB in the figure), it is slightly coarser and partially discontinuous, while in the heat-affected region between consecutive layers (HAZ in the figure), it is almost entirely interrupted. Therefore, in the as-built condition, the peculiar microstructure is extremely inhomogeneous and closely dependent on the process parameters, such as building direction and scanning strategy. The T5 alloy was still characterized by the extremely fine cellular structure in the as-built condition (Figure 7b,e). However, in some regions, the eutectic-Si network appeared slightly fragmented, less defined, and thinner than the as-built one. Finally, the T6R treatments deleted any trace of the manufacturing process (Figure 7c,f). As a result, the eutectic-Si network completely broke down, and the microstructure consisted of a fine distribution of globular and sub-micrometric Si-rich particles dispersed in the α-Al matrix. T6R treatment also succeeded in homogenizing the microstructure, as no difference in morphology was evidenced between the xy and the xz planes.

The definition of strengthening mechanisms involved in the Al-Si-Mg PBF-LB and their relative contribution is still an open field of research, even if several attempts have been made to correlate tensile behavior to microstructure [9]. However, according to the most recent literature, Si plays a significant role in defining the mechanical properties of the alloy, and it can be found in the form of: (i) solid solution; (ii) nanometric-sized Si-particles dispersed within α-Al cells; (iii) eutectic-Si network. Consequently, based on both mechanical and microstructural analyses, as well as literature findings [47,48,49,50], it can be assumed that the main strengthening mechanisms involved in the Al-Si-Mg PBF-LB alloys are: (i) microstructure refinement and solid solution in the as-built condition; (ii) microstructure refinement, solid solution, and precipitation of nanometric-Si from the supersaturated solution in the direct aged (T5) condition; (iii) precipitation of both nanometric-Si and Mg_2_Si precursors strengthening phases in the T6R condition. Previous studies on the role of post-process heat treatments of the PBF-LB AlSi7Mg alloy carried out by the authors [25] confirmed that partial precipitation of Si from the supersaturated Al matrix occurred after direct aging. However, more prominent precipitation of Si occurred after the solution treatment and, after artificial aging, also Mg_2_Si precursors were detected by XRD analyses. Hence, precipitation strengthening justifies the increase in the yield stress observed for T5 and T6R.

Furthermore, the role of residual stress should also be considered, which can strongly affect mechanical behavior. In the as-built condition, samples were characterized by tensile residual stress, only partially removed by T5 treatment. T6 treatment, on the other hand, succeeded in completely relieving tensile residual stress and presumably induced slight compressive stress [25]. Although, it is worth mentioning that, especially in the case of tensile strength and ductility, strengthening mechanisms and residual stress are not the only ones responsible for defining mechanical properties. Microstructural defects, like porosities, can be detrimental and affect the structural integrity of the alloy. Therefore, the overall mechanical behavior is balanced between these three fundamental aspects.

#### Work Hardening

**Figure 8:**
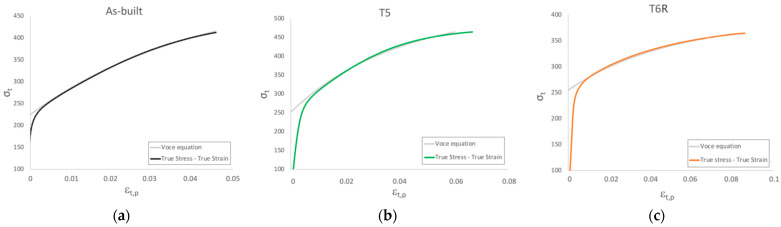
Comparison between true stress-true strain experimental curves and flow curves obtained with Voce equation of representative: (**a**) as-built, (**b**) T5, and (**c**) T6R tensile samples.

Significant differences among the temper conditions were found in the work-hardening behavior. Further analyses were conducted on the plastic region of true stress-strain curves to correlate it to the microstructural features. In the literature, the analysis of the plastic region of stress-strain curves is used to assess the structural integrity of the alloy as the presence of defects, like internal material discontinuities, affects the plastic behavior of the alloy [36,37,54]. While yield stress is marginally affected by defects, Rm and A% strongly depend on it [33]. In the case of PBF-LB, parts are characterized by specific internal defects, such as gas porosities and lack of fusions. Their effect on the mechanical response should be considered. Therefore, the Voce equation and Kock-Mecking model [55,56] investigated the work-hardening behavior of as-built, T5, and T6R samples. Voce equation (Equation (1)) was developed to describe the plastic flow behavior, and it is more effective in the case of fcc-metals than other well-known laws such as Hollomon, Ludwik, and Ludwigson ones [57]. As shown in Figure 8, the Voce equation can be proficiently used also to describe the behavior of the AlSi7Mg PBF-LB alloy.

Voce equation is expressed as:

(1) σt=σs+(σs+σ0)−(εt,pε0)

where$\sigma_{s}$ is the saturation stress in case of full plasticity, reached when work hardening rate θ=dσε=0; $\sigma_{0}$ is the threshold stress reached when the true plastic strain $\varepsilon_{t,p}$ = 0; $\varepsilon_{0}$ is the strain characteristic of the Voce equation controlling the shape of the curve. 

**Figure 9:**
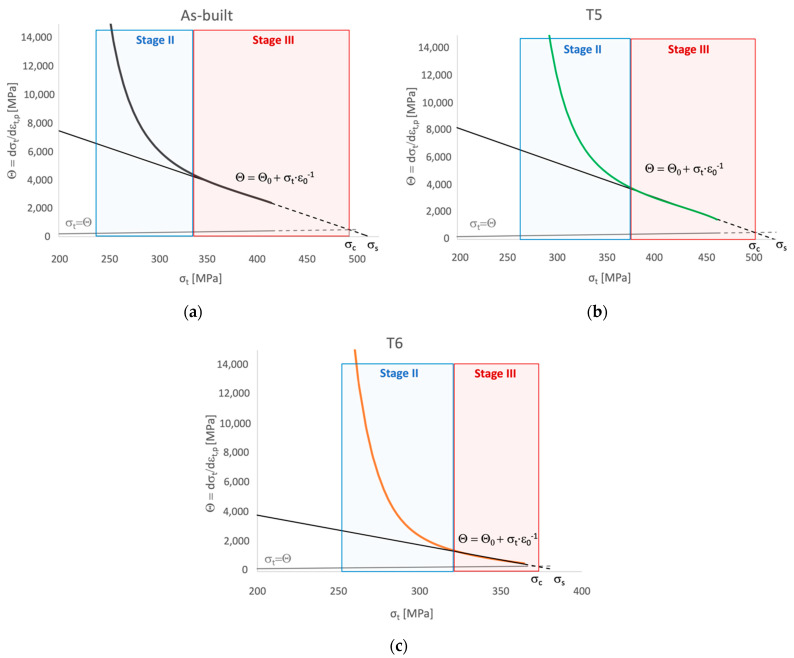
Kocks-Mecking (KM) diagram obtained for the AlSi7Mg PBF-LB alloy in the: (**a**) as-built (black line), (**b**) T5 (green line), and (**c**) T6R temper condition (orange line). Solid lines represent the elaboration of experimental data from tensile tests; dashed lines represent linear extrapolation from experimental data.

Based on the Voce equation, the Kocks-Mecking model (Equation (2)) was developed to describe the Stage III of work hardening, characterized by a linear behavior in a diagram representing the work hardening rate θ as a function of the true stress $\sigma_{t}$, termed as Kock-Mecking (KM) diagram (Figure 9).

The Kocks-Mecking model is expressed as:

(2) θ=dσdε=θ0+Kσt

where $\theta_{0}$ work hardening rate when σ=0 and is a parameter mainly depending on Stage II work hardening rate and strain rate. According to Angella et al. [54], this parameter can be obtained based on the Voce equation as . From a physical point of view, $\theta_{0}$ is an athermal constant that describes the dislocation storage rate and is inversely related to the characteristic dimension of the microstructure, while K is a thermal parameter related to the dynamic recovery [54,55,57].

Work hardening behavior of polycrystalline metals can be divided into three stages: (i) non-linear Stage II, where athermal work hardening occurs at a high rate; (ii) linear Stage III, where work hardening and softening mechanisms compete, this region is temperature, and strain-rate sensitive as thermal activation facilitate the dynamic recovery; (iii) non-linear Stage IV where tensile instability is reached. Fracture of samples without major internal defects affecting their structural integrity occurs during Stage IV [37,58,59]. In the same diagram, also the equation characteristic of the onset of the diffusive necking phenomenon θ=dσdε=σt, according to the Considère criterion σt,necking=(dσtdεt,necking), can be represented [60,61]. Therefore, the intersection between the Stage III linear equation and the necking one, which occurs at $\sigma_{t}=\sigma_{c}$, can be considered as the threshold between Stage III and Stage IV. As depicted by KM diagrams in Figure 9, samples did not reach Stage IV. Therefore, fracture occurred within Stage III, suggesting a major influence of internal defects in the tensile behavior. Only in the case of the T6R condition, experimental data (solid lines in Figure 9) covered almost entirely Stage III.

**Figure 10:**
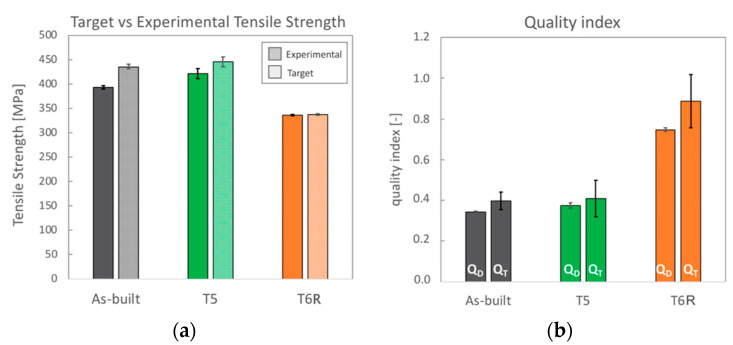
Results from the analyses of the strain hardening behavior AlSi7Mg PBF-LB alloy in the as-built, T5, and T6R temper condition: (**a**) experimental tensile strength obtained from tensile tests compared to the target one; (**b**) quality index (Q_D_ index is based on ductility, Q_T_ index is based on toughness).

This result was also confirmed by comparing the experimental R_m_ values for each temper condition (already discussed in Figure 4) to the target one, mathematically obtained following the procedure proposed by Tiryakioglu et al. [36] and reported in Figure 10a. As a result, variations among samples are quite reduced. Furthermore, comparing experimental and target data that could be reached in case of the absence of detrimental internal discontinuities shows that only T6R samples reached R_m_ values close to the target one. Since samples were produced with the same processing conditions, it can be assumed that a comparable content of internal defects characterized them. Therefore, the analysis conducted here suggests that the modification in the microstructure due to the T6R treatment reduced the detrimental effects of internal defects in the plastic behavior of the AlSi7Mg PBF-LB alloy.

Analogous results were obtained by comparing quality indexes (Figure 10b). The indexes here considered were elaborated based on the analysis of strain hardening behavior and structural integrity of conventional Al alloys [36,37,38,54] and derived from the ductility (*Q_D_*) and toughness (*Q_T_*) of the alloy. The quality index *Q_D_* (Equation (3)), also known as the relative ductility parameter *q* [38], compares the theoretical uniform strain up to the onset of the necking phenomenon $\varepsilon_{unif}$, calculated as proposed by Angella et al. [54], and the strain at fracture $\varepsilon_{f}$ obtained by tensile tests. $\varepsilon_{unif}$ represents the maximum practical ductility of the material; therefore, materials that undergo failure before reaching this value (*Q_D_* < 1) suffer from internal discontinuities that affect their ductility. The quality index *Q_T_* (Equation (4)), proposed by Tiryakioglu et al. [36], compares the target toughness value $\psi_{c}$, that has to be reached during the tensile test in a sample free from major discontinuities, and the toughness ψ obtained from experimental tensile test. Toughness can be evaluated from the area under the stress-strain curves, as in the present work, or mathematically as a function of the values R_p0,2_, R_m,_ and A%. *Q_T_* index was developed on the concept that the adsorbed energy, thus the toughness, is directly related to the effective crack length produced by discontinuities. Therefore, samples characterized by major internal discontinuities will return *Q_T_* < 1.

(3) QD=εunifεf

(4) QT=ψCψ

*Q_D_* and *Q_T_* indexes obtained for the AlSi7Mg PBF-LB alloy in the as-built, T5, and T6R conditions were lower than 1, thus indicating that internal defects affected the mechanical response of the alloy. However, the T6R state showed the highest quality index, thus confirming that the modifications induced in the microstructure by the tailored T6R treatment, probably also in relieving residual stress as found in previous work [25], reduced the detrimental effect of internal defects.

As previously mentioned, parameters of the Voce equation describing Stage III of strain hardening are closely related to microstructural features. For example, *Θ*_0_ is inversely proportional to the mean free path of mobile dislocation [39]; therefore, it can be correlated to the finesse of the microstructure, like, in the case of PBF-LB, cells dimensions, grain size, and size of Si nanoparticles. On the other hand, *ε*_0_^−1^ is related to dislocation motion, and, in particular, it depends on the crystallographic lattice in which dislocations move [39]. Therefore, a great value of *Θ*_0_ means a fine microstructure and a great *ε*_0_^−1^ value indicates a high tendency to recover dynamically. The strain hardening behavior in Stage III depends on the balance between hindering of dislocation motion and dynamic recovery, so high *Θ*_0_ and *ε*_0_^−1^ are related to high strain hardening ability. The parameters of the Voce equation obtained in the present study are summarized in Table 4. Based on the above, as-built and T5 temper conditions were characterized by the highest values of Voce parameters, thus indicating a very fine microstructure and a significant tendency to strain hardening. In particular, the T5 temper condition was able to maximize both parameters. In the case of T6R, the *Θ*_0_ value was reduced by approximately 50%, suggesting that the heat treatment induced a significant coarsening of the microstructure. On the contrary, the *ε*_0_^−1^ value was comparable to the as-built one, thus suggesting that the ability for dynamic recovery was not affected by the T6R treatment.

**Figure 11:**
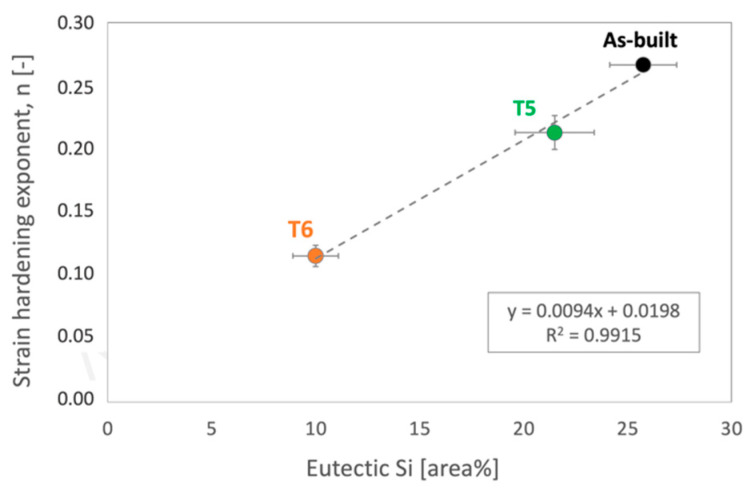
Correlation between strain hardening exponents n measured from true stress-true strain experimental curves and area% of the Si-rich region evaluated on FEG-SEM micrographs for the as-built, T5, and T6R AlSi7Mg PBF-LB tensile samples.

Accordingly, strain hardening exponents, *n*, were evaluated for all the alloy conditions. Results showed that the as-built alloy was characterized by the highest strain hardening exponent (*n* = 0.26 ± 0.004), which decreased after both T5 (*n* = 0.21 ± 0.01) and T6R (*n* = 0.11 ± 0.01) treatments. This result partially confirmed the above outcome and was obtained from the Voce equation. It is known that a change in the strain-hardening behavior suggests a modification in the type or dimension of microstructural features hindering dislocation motion, mostly related to the presence of Si [9,47,48,49,50]. In support of these findings, a strong correlation was found in the present work by comparing the area percentage occupied by the eutectic-Si network in as-built and T5 alloy, or Si-rich globular particles in the T6R one, with the hardening exponent *n* (Figure 11). Area percentage was measured by image analyses of FEG-SEM micrographs analogous to those reported in Figure 7. By moving from the as-built to the heat-treated condition, the Si-rich area% decreased. Presumably, in the T5 condition, this is due to the shrinkage of the eutectic-Si network because of the precipitation of nanometric Si particles from both the supersaturated matrix and the eutectic network. After T6R treatment, complete precipitation of Si from the supersaturated matrix occurred, along with precipitation of Mg_2_Si reinforcing phase precursors [25]. These precipitates are appreciable only at the nanometric scale, so they were not detected by FEG-SEM analyses. As a result, the area% of the Si-rich region after T6R treatment decreased to approximately the 30% of the area occupied by the eutectic-Si network in the case of as-built alloy. Nevertheless, the strong correlation in Figure 11 suggests that the thick and fine eutectic-Si network surrounding the α-Al cells, acting as a two-phase aggregate strengthening mechanism, is primarily responsible for the great strain hardening behavior of the as-built and T5 AlSi7Mg PBF-LB alloy. In the case of T6R alloy, where the eutectic-Si network is no longer present, and the strain hardening behavior is considerably reduced, different microstructural features (i.e., sub-micrometric Si-rich particles or nanometric reinforcing particles) hinder dislocation motion, presumably in relation to precipitate cutting and precipitate looping mechanisms.

### 3.2. High-Temperature Tensile Behavior: Effect of Thermal Exposure

When tested at 200 °C, despite the temper condition, samples showed comparable tensile properties (both mean values and standard deviations, Figure 4 and Figure 5). The marked difference in the strain-hardening behavior observed at room temperature was considerably reduced (Figure 3). Based on the previous discussion, it can be argued that the exposition at high temperatures during tensile tests minimized the differences in the structural integrity among temper conditions that, conversely, strongly affected the room temperature behavior. As expected, due to the improved mobility of dislocations at high temperatures, yield stress and tensile strength decreased compared to room temperature tensile tests [23,62]. Accordingly, also Young’s modulus decreased from approx. 70 to 50 GPa as a consequence of the exposure to high temperature during the test. The yield stress ranged between 172 and 183 MPa, maximum in the case of as-built samples, while the tensile strength was 209 MPa for as-built and T5 conditions and 187 MPa for T6R. Ductility, on the other hand, for as-built and T5 conditions increased up to 10%, doubling the values obtained at room temperature, while in the case of T6R, it slightly decreased from 9.7 ± 1.8 to 8 ± 1.0%. In terms of modulus of resilience and toughness (Figure 5b,c), the as-built condition was able to preserve the values obtained at room temperature; therefore, its ability to adsorb energy in the elastic and plastic region was not affected by the exposure at high temperature during the tensile test.

**Figure 12:**
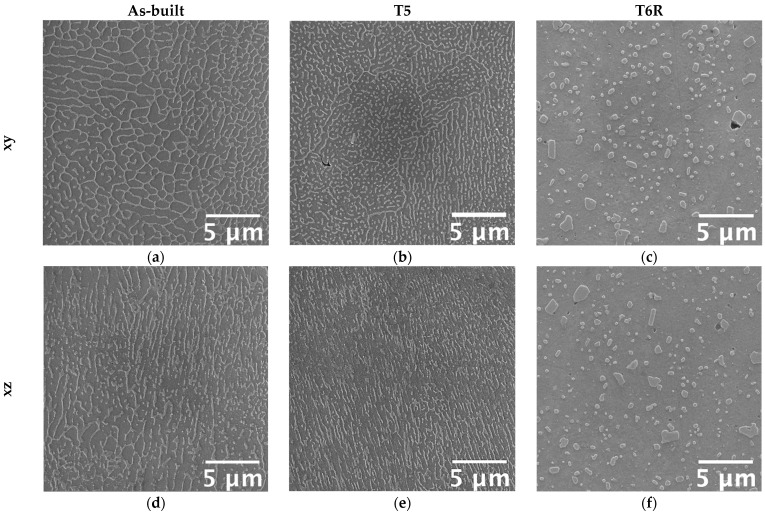
FEG-SEM high magnification micrographs showing the typical microstructure of the as-built, T5, and T6R AlSi7Mg PBF-LB alloy after tensile tests performed at high temperature (200 °C): (**a**–**c**) along the direction parallel to the building platform (xy plane); (**d**–**f**) along the direction parallel to the building one (xz plane).

On the other hand, this ability was strongly affected in the T6R condition, which showed a significant decrease in both moduli (−40% for U_R_ and −56% for U_T_). T5 condition exhibited a lower resilience (−47% for U_R_) but a higher toughness (+28% for U_T_). At the temperature of 200 °C, the as-built condition showed the best overall mechanical behavior, possibly related to its supersaturated solid solution condition. In fact, by observing the residual hardness measured after the test and by comparing it to the values measured before the test (Figure 6), only the as-built condition could increase the hardness after tests. This result presumably relates to the precipitation of nanometric Si particles consequent to the exposition at high temperatures during tests. By observing the microstructure (Figure 12), both as-built and T5 conditions still presented the typical cellular microstructure after high-temperature tests. Compared to the micrographs in Figure 7, it can be noticed that a slight fragmentation occurred due to the high-temperature exposition, as described in [24], even if the phenomenon is more accentuated in the T5 (Figure 12b,e) condition than as-built (Figure 12a,d) one. Mainly if observed on the xy section, the eutectic-Si network on T5 (Figure 12b) samples was almost entirely interrupted, while an overall continuity was maintained on as-built (Figure 12a) ones. Anisotropy in the cellular structure among xy and xz sections was still present in both cases. At the investigated magnification, the T6R microstructure (Figure 12c,f) did not show significant differences due to the high-temperature exposure compared to the microstructure before tests (Figure 7c,f).

### 3.3. Fractographic Analysis

**Figure 13:**
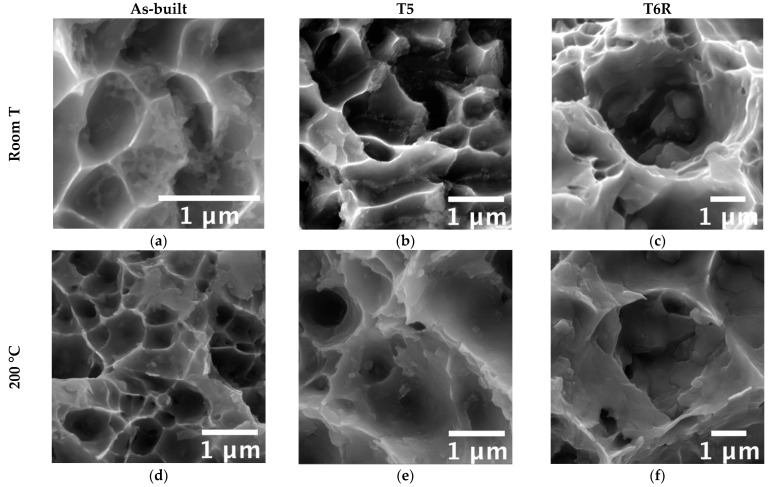
FEG-SEM high magnification fractographic analyses of AB, T5, and T6R AlSi7Mg PBF-LB tensile samples after (**a**–**c**) tests performed at room temperature; (**d**–**f**) tests performed at high temperature (200 °C).

A ductile fracture mode characterized all tested conditions. High magnification analyses of fracture surfaces (Figure 13) revealed the presence of very fine dimples that denote a ductile fracture. Dimples form due to material yielding and usually nucleate in correspondence with material discontinuities. Different microstructural features could be recognized inside dimples based on the temper condition. On as-built samples (Figure 13a,d), a trace of the eutectic-Si network was evidenced, especially on samples tested at room temperature (Figure 13a), as also observed by Casati and Vedani [12]. After the high-temperature exposure, sub-micrometric particles could be found inside dimples (Figure 13d), confirming the occurrence of Si precipitation from a supersaturated solution. Similarly, sub-micrometric particles were observed inside dimples on T5 samples (Figure 13b,e). By supporting the above-discussed microstructural analyses, traces of the continuous cellular structure were evident only on room-temperature tested samples (Figure 13e).

**Figure 14:**
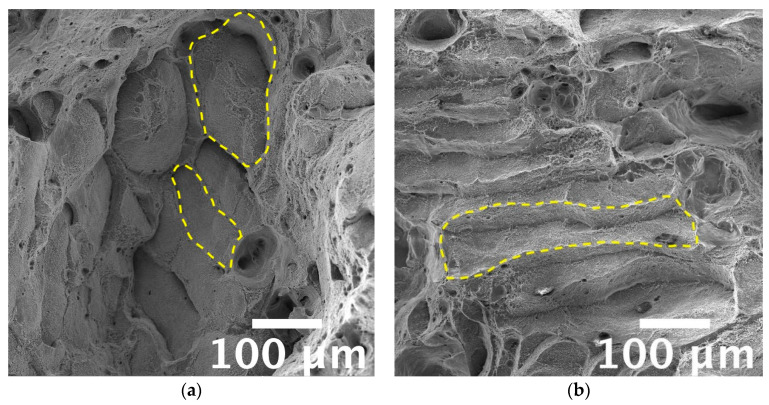
FEG-SEM high magnification fractographic analyses, detail of successive layers de-cohesion on (**a**) AB, (**b**) T5 AlSi7Mg PBF-LB tested at 200 °C. Yellow dashed lines highlight some features related to laser scan tracks.

**Figure 15:**
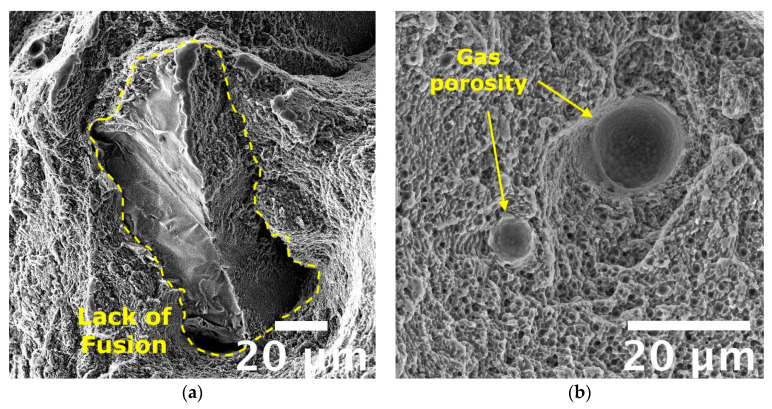
FEG-SEM high magnification fractographic analyses of the AlSi7Mg alloy tensile samples show the main defects affecting mechanical behavior: (**a**) lack of fusion and (**b**) gas porosities.

Furthermore, Si particles were found inside dimples in the T6R alloy (Figure 13c,f) that, in most cases, appeared fractured. Due to the flat surfaces of broken Si particles, it can be inferred that a brittle fracture mechanism occurred for them, as also observed by Trevisan et al. [16]. Accordingly to microstructural observations, the finest dimples were observed for the as-built and T5 alloys because of the fine cellular microstructure observed in these conditions. The absence of appreciable microstructural inhomogeneities, as Si-rich particles formed after T6R, delayed dimples nucleation and hindered their growth, as confirmed by the overall fine dimples dimension (approx. 1 µm in the case of as-built alloy). These outcomes agree with the previously discussed tensile results in which both as-built and T5 samples showed greater ultimate tensile strength and hardening than T6 samples. Such finesse was still preserved by the as-built alloy tested at high temperatures, supporting the good overall mechanical behavior discussed in the previous section. It is worth mentioning that only in the case of tests conducted at high temperatures on as-built and T5 samples it was possible to recognize traces of laser scan track on fracture surfaces, as evidenced in Figure 14. Such features evidence the de-cohesion of successive processed layers in correspondence with HAZ regions [63]. Other authors also observed this feature on samples built perpendicularly to the platform, as in the present work [64,65]. However, different from this study, the literature work adopted a platform pre-heating (150–200 °C) for the PBF-LB process and evidenced the presence of scan tracks on surfaces of samples tested at room temperature. Therefore, the de-cohesion of successive processed layers may be promoted by microstructural modifications induced by exposure to high temperatures during the process or testing. Lastly, the primary defects of this alloy affecting the mechanical behavior, and thus the structural integrity, are represented in Figure 15. Defects found on fracture surfaces of tensile samples, regardless of temper condition or testing temperature, can be divided into two main categories: (i) large and irregular lack of fusion, with dimensions usually greater than 100 µm and an oxidized surface (Figure 15a, highlighted in yellow dashed line); (ii) spherical gas porosities with dimension in the order of tens of micrometers (Figure 15b, highlighted by yellow arrows).

## 4. Conclusions

The present work aims to characterize the tensile properties at room and high (200 °C) temperature of the A357 (AlSi7Mg0.6) PBF-LB alloy obtained with no platform pre-heating in the as-built condition and after two dedicated heat treatments at peak-aging: direct aging (T5) and rapid solution treatment, quenching and aging (T6R). First, tensile results were discussed based on both fractographic and microstructural analyses. Concurrently, the effect of typical PBF-LB microstructural defects on the structural integrity of the alloy for each heat treatment condition was evaluated by analyzing the work hardening behavior. Based on the results, the role of microstructural features and defects on tensile behavior can be synthesized as follows:

- As-built alloy showed good overall mechanical behavior at both room and high temperatures, thanks to the distinctive microstructural features (i.e., supersaturated solid solution, cellular structure composed by sub-micrometric α-Al cells surrounded by eutectic-Si network and dispersion of nanometric-sized Si-particles within α-Al cells;) that induced microstructural refinement, solid solution, and two-phase aggregate strengthening mechanisms and high work hardening at room temperature. However, the alloy showed the highest sensitivity to the presence of defects, which strongly affected the tensile response of the alloy.
- T5 alloy preserved the peculiar cellular structure of the as-built alloy along with its strengthening mechanisms and high work hardening. Moreover, due to the induced precipitation strengthening, yield stress and tensile strength increased concerning the as-built alloy without a significant decrease in ductility. Therefore, the resilience of the alloy was enhanced without affecting its toughness. At the same time, when exposed to high temperature, T5 alloy could preserve satisfying mechanical properties, comparable to the as-built one. The sensitivity of the T5 alloy to the presence of defects was slightly lower than the as-built one. However, the structural integrity was still strongly affected by defects.
- T6R alloy was characterized by a coarser but more homogeneous microstructure composed of globular Si-rich particles dispersed in the Al matrix with no trace of cellular structure. T6R resulted in the best trade-off between strength and ductility, with precipitation as the primary strengthening mechanism, and reduced work hardening. Moreover, T6R enhanced the structural integrity of the alloy, which showed an almost negligible sensitivity to internal defects. However, when tested at high temperatures, T6R exhibited the lowest mechanical properties among the tested temper conditions, even if the results are still comparable to as-built and T5 alloys.

Results indicate that post-process heat treatments tailored to the peculiar Al-Si-Mg PBF-LB microstructure can tune the mechanical response, especially at room temperature. Concurrently, for a comparable defect content, the heat treatment positively reduces the detrimental effect of internal defects (e.g., lack of fusion and porosity) on the mechanical behavior. Therefore, both aspects should be considered when designing structural components. However, despite differences evidenced by microstructural analyses, minor deviations in the tensile properties were found among as-built, T5 and T6R conditions when tested at high temperatures. Given the possible application in the automotive or motorbike industry of Al-Si-Mg alloys, especially the AlSi7Mg0.6 one, for components in the power train system, analysis dedicated to the thermal stability will be addressed further in future works.

## Figures and Tables

**Table 1:** The powder’s chemical composition (wt.%), given by the supplier [29], and PBF-LB tensile samples were checked by GDOES.

	Al	Si	Mg	Fe	Cu	Mn	Ti	Zn	O	N	Other
Powder	Bal.	6.90	0.55	0.09	<0.05	<0.01	0.09	<0.01	0.1	<0.2	<0.1
PBF-LB tensile samples	Bal.	7.34	0.56	0.08	-	-	0.14	0.07	-	-	0.02

**Table 2:** PBF-LB process parameters used for tensile sample manufacturing.

Support Type	Laser Power [W]	Scan Speed [mm/s]	Layer Thickness [μm]	Hatch Space [μm]	Scan Strategy
Cones	175	500	20	80	3 × 3 mm^2^ chessboard

**Table 3:** Heat treatments (optimized in [25]) and tensile test conditions.

	Heat Treatment Conditions					Tensile Test Temperature	
	Solution Treatment		Water Quenching	Artificial Aging			
	T [°C]	t [min]		T [°C]	t [h]		
As-built	-	-	-	-	-	Room T	200 °C
T6R	540	10	Warm water (60 °C)	150	4	Room T	200 °C
T5	-		-	170	1	Room T	200 °C

**Table 4:** Parameters of Voce equation, *Θ*_0_ and *ε*_0_^−1^, obtained by the analysis of strain hardening behavior of the AlSi7Mg PBF-LB alloy tested in the as-built, T5 and T6R condition.

Temper Condition	*Θ*_0_ [MPa]	*ε*_0_^−1^ [-]
As-built	12.1 ± 0.7 × 10^3^	23.9 ± 1.6
T5	16.4 ± 2.1 × 10^3^	32.3 ± 4.7
T6R	8.0 ± 0.6 × 10^3^	20.4 ± 1.7

## Data Availability

The raw/processed data required to reproduce these findings cannot be shared as the data also forms part of an ongoing study.
